# Translational research in health technologies: A scoping review

**DOI:** 10.3389/fdgth.2022.957367

**Published:** 2022-08-03

**Authors:** Nadja N. V. Mayrink, Luís Alcoforado, Arthur Chioro, Felipe Fernandes, Thaisa S. Lima, Erika B. Camargo, Ricardo A. M. Valentim

**Affiliations:** ^^1^^ Centre for Interdisciplinary Studies, Institute for Interdisciplinary Research, University of Coimbra, Coimbra, Portugal; ^^2^^ Laboratory of Technological Innovation in Health (LAIS), Federal University of Rio Grande do Norte (UFRN), Natal, Rio Grande do Norte, Brazil; ^^3^^ Faculty of Psychology and Education, University of Coimbra, Coimbra, Portugal; ^^4^^ Escola Paulista de Medicina, Departamento de Medicina Preventiva, Universidade Federal de São Paulo, São Paulo - SP, Brasil; ^^5^^ Brazilian Ministry of Health (MoH), Brasília, DF, Brazil; ^^6^^ Evidence Program in Policies and HealthTechnologies, Oswaldo Cruz Foundation (FIOCRUZ/Brasília), Brasília, Brazil

**Keywords:** medical research, translational research, medical device, health innovation, health technologies

## Abstract

**Introduction:**

The current debate on the process of technological innovation points out as a challenge for universities consolidation of competencies that allow the generation and transfer of knowledge to society. The Translational Research (TR) approach has as one of its main objectives the acceleration of the innovation process, based on the transposition from basic science to applied science and innovation, which comprises the different stages of research, development and innovation. The literature points out that the dynamics of translation, which results in new technologies, are complex, transdisciplinary, inter-institutional, systemic, and non-linear. The main objective of this review is to contribute to the adoption of institutional strategies and the formulation of public policies aimed at solving today’s social and economic challenges, ensuring access to technologies and sustainability for the health system. The specific objectives were: (i) to systematize studies that characterized translational research in medical devices; (ii) map the challenges for the implementation of translational health research; (iii) contribute to the design of institutional strategies; and (iv) support the formulation of public policies.

**Methods:**

This study used the scoping review technique, according to PRISMA-ScR and the Joanna Briggs Institute guidelines. Concerning the extraction of relevant articles, the journals indexed in Bireme, Pubmed, Scopus, Web of Science, and Google Scholar were consulted for selecting relevant articles. The search was carried out on November 28, 2021, updated on April 29, 2022, and there were no restrictions as to the year of publication, language or type of analysis. Studies that did not answer the research question were excluded, as they dealt exclusively with the pharmaceutical segment, the translation of knowledge into clinical practice, or addressed the process of translational research applied to specific diseases or technologies.

**Results:**

Thirty-three articles were included indicating that the approach of translation of research is multidisciplinary and transdisciplinary and encompasses knowledge and aspects that go beyond basic and applied research and incorporates final steps concerning regulatory aspects, clinical research, market analysis, technology transfer, production and incorporation of technologies into the health system.

## Introduction

The contemporary debate on technological innovation, which integrates the development of new products or processes in the productive and social environment, presents challenges for the academic area, particularly in scientific and technological research (1–4). In this context, it is necessary to develop innovative competencies that allow the translation, i.e, the generation and transference of the knowledge produced in the academy to society. The focus on knowledge generation, diffusion and interaction supports the concept of the National Innovation System (NIS).

The NIS is characterized by the articulation of public and private sector institutions, whose activities and interactions generate, adopt, modify and disseminate new technologies. Such interaction occurs from the flows of knowledge generated, its material resources, regulations and local policies, in order to support technological development (5–7).

In the health area the literature also treats the innovation process from the “translation” approach, which emphasizes the systemic and dynamic character of the knowledge production process (8–11). “Knowledge translation” differs from “translation of knowledge” because it establishes flows in various directions, based on the dialogue and production of knowledge among different actors (12). This multidirectional characteristic is evidenced in the path of development of health technologies, from their invention to their availability to society: a non-linear path that, in many cases, complies with simultaneous stages in the translation process. They become even more challenging because they are technologies, in general, that have an incremental and non-substitutive character, which makes their incorporation by health systems even more complex and costly.

The Translational Research (TR) approach originated in the USA in the 1990s to promote interdisciplinarity and accelerate the innovation. It considers the transposition from basic to applied science, which comprises the different stages of research, development and innovation (12).

In its analyses, the translational approach incorporates factors related to the innovation cycle, including the final stages related to clinical research and the regulation and supply of health products and services (6). These specificities favor that translational research is better understood in the conceptual framework of the NIS (13–15), which in Brazil also refers to the approach of the Health Economic-Industrial Complex (HEIC) (16–19).

Gadelha et al. (16) describes the HEIC as the permanent dynamics of interdependence and interaction between society and the State, including technological and industrial sectors, universities, and health services searching for the supply of health services and products. The HEIC incorporates the complex nature of innovation in health, which, in turn, integrates tangible components, such as medications, vaccines, and medical devices, and intangible elements, such as information systems to support diagnosis and treatment, procedures, therapies, and digital health solutions (18,20). Through the characterization of all these health innovation components by developing technologies associated with health care anemphasizing the health system.

Thus, the innovation cycle in health includes both tangible and intangible technologies. In other words, the cycle is not restricted to a type of technology and ordered succession of stages, nor to a set of fixed requirements common to all technologies in health. This perspective presents a relevant potential for the public health system, as it is an important demand of solutions for its different tensions, which the general buying power can enhance. These tensions come from the market, health professionals, users, and health system managers. Thus, translational research also means translating knowledge into practical actions in the political and organizational dimensions (13, 21).

According to the World Health Organization (WHO), health technology is the “application of knowledge and skills organized in devices, drugs, vaccines, procedures, and systems developed to combat a health problem and improve life quality.” (22).

In Brazil, the development of non-tangible technologies applied to health occurs increasingly. The implementation of the National Registry of patients with Amyotrophic Lateral Sclerosis (ALS) and other technologies in the context of ALS (23–25), the set of systems for epidemiological surveillance, regulation of medical beds, vaccination toward the Covid struggle (26), and the platform for human training in health, the AVASUS (27) are examples of interdisciplinary research in intangible technologies applied to health in Brazil.

On the other hand, the development of medical equipment advances at a different pace and frequency. The repository of the National Institute of Intellectual Property (INPI) shows, in 2021, the filing of 41 computer programs for the human health field and 17 patents related to equipment and devices for use in health linked to Brazilian universities and companies established in the country (28).

There is a gap between Brazilian data on scientific production and innovation in health. The last decades were marked by a significant growth in scientific production, which went from 20 to 182 publications per million inhabitants between the 1990s and 2013. This growth was also evidenced in the increase in the Brazilian percentage participation in world publications, which jumped from 0.7% to almost 3% in this same period. However, the percentage of Brazilian companies that created genuinely new products or processes in the domestic market, remained at 4% in the same period (29).

The medical devices segment, which comprises a broad set of products, equipment, devices, materials, articles or systems for use or application in health, intended for prevention, diagnosis, treatment, rehabilitation or contraception (30). The world’s leading companies allocate between 9% and 10% of their annual revenues in research and development (R&D) and most of these resources are directed to the improvement of existing products rather than the introduction of new technologies (31). Companies installed in Brazil showed in 2017 a ratio of 1% between spending on internal R&D activities and net revenue, as shown in the Industrial Research of Technological Innovation (Pintec) (32).

In this context, it is essential to qualify the factors that permeate innovation in health and its specificities in translational research in medical devices.

This review aims to systematize studies that characterize translational research in health technologies and the challenges to its implementation. It intends to contribute to the strengthening of health innovation in Brazil, specifically for the formulation of more effective public policies and the adoption of timely institutional strategies in the face of current social and economic challenges.

The main objective of this review is to contribute to the adoption of institutional strategies and the formulation of public policies aimed at solving today’s social and economic challenges, ensuring access to technologies and sustainability for the health system. The specific objectives were: (i) to systematize studies that characterized translational research in medical devices; (ii) map the challenges for the implementation of translational health research; (iii) contribute to the design of institutional strategies; and (iv) support the formulation of public policies.

## Methods

For this study, the scoping review technique was adopted, according to the Joanna Briggs Institute guidelines (33). The methodology was structured in six stages: (1) formulation of the research question and objectives; (2) preparation of a protocol containing the search strategy and inclusion criteria; (3) search in repositories and electronic indexers, which would enable the breadth and scope of this scoping review; (4) selection of studies by independent evaluators, through the Rayyan platform, according to the inclusion criteria pre-defined in the protocol; (5) summarization of the results, based on qualitative analysis in relation to the objectives and research question; (6) presentation of the results and analysis of the implications in the adoption of strategies for the development of translational research in health technologies.

To formulate the research question, the acronym SPiDER (Sample, Phenomenon of Interest, Design, Evaluation and Research Type) was used as support. This scope review is configured as a qualitative study, which composes its sample of studies that addressed the theme, eligible in the review, being the phenomenon of interest in translational health research. In the health technologies field, we chose to focus on the medical devices segment, in order to delimit the scope and refine the search. The question formulated was: “What are the characteristics, factors and dimensions that integrate translational research in medical devices and the challenges for its implementation?”

After the question was elaborated, the descriptors and keywords were identified to capture the articles referring to the theme of this study, namely: “Translational Medical Research” OR “Translational Research” AND “Medical Device” AND “Invention” OR “Innovation.”

### Record of the protocol

The scoping review protocol has been registered on the Open Science website under the number cn63y available at: https://osf.io/cn63y/.

### Eligibility Criteria

The inclusion criteria were case studies, systematic reviews, or other types of reviews that addressed characteristics of translational research in medical devices, with no language restrictions or publication date limits.

The exclusion criteria were studies that did not fully or partially answer the review’s guiding question, such as studies that addressed the translation of knowledge into clinical practice, that addressed characteristics exclusively of the pharmaceutical segment, or that addressed translational research applied to specific diseases or technologies.

### Information sources (databases)

To identify relevant studies, the journals indexed in Bireme, PubMed, Scopus, Web of Science, and Google Scholar were consulted. These databases were selected because they are comprehensive, having wide coverage of publications in the health area and in the interdisciplinary field. Manual searches were performed for articles with potential for inclusion in the present study.

### Search Strategy

The search was performed November 28, 2021, updated on April 29, 2022, and took place in four stages:

1. Initial search limited to two online databases appropriate and relevant to the topic. The databases were Scopus and Pubmed. This initial search served to analyze the text keywords contained in the title and abstract of the retrieved articles and the index terms used to describe the articles;
2. Search conducted in all included databases (Bireme, Scopus, Web of Science, Pubmed, and Google Scholar), from all keywords and index terms identified in step 1. The authors independently screened the titles and abstracts (first-level screening) of retrieved articles, excluding repeated articles, to establish the eligibility of articles that met the inclusion criteria for analysis. All articles that satisfied the first-level screening were retained for second-level screening (full-text article review). Again, the authors independently screened full-text articles to determine inclusion in the scoping review; and
3. Search containing the reference list of reports and articles identified as additional sources of the studies that were included in the review.
4. The search strategy relied on the keywords: “Translational Medical Research” AND “Equipment and Supplies” AND “Innovation” as a starting point, the sensitive search strategy is in **Supplementary Material 1**.

### Study selection process

The studies identified by the searches performed in the previously mentioned databases were entered into Mendeley and then independently evaluated in Rayyan.

### Data Extraction and Synthesis

The studies selected to compose this review were mapped using an Excel® spreadsheet with the following information: author(s), year of publication, title, country of origin, type of study, and excerpts describing the results of interest to this review.

In the summarization stage, from the qualitative analysis of the data, the information was categorized as: characteristics (i), referring to the aspects and attributes inherent to translational research; barriers (ii), as difficulties and adversities faced by academic laboratories in the process of translational research; and factors (iii), which comprise the conditions that favor the development of translational research related to medical devices. We identified three dimensions of common understanding to the categories from this categorization, which contribute to the elucidation of the most relevant aspects for this review. They are: the knowledge dimension (1), referring to the content that can be learned; the capacity dimension (2), which is associated with knowing how to do, indicates an acquired skill; and the dimension relative to the institutional or inter-institutional structure (3), physical or management conditions of an institution or a set of institutions. This methodological option was made due to the nature of the process studied in the included articles and the potential contribution of this analysis to the understanding of the research problem (33–35).

The calculation of incidence (i) in the tables of results was performed from the identification in percentage of the number of times that a certain theme or concept (q) appeared in the total number of articles included in this review (t), as shown below:

(1) $$i=\left(\frac{q}{t}\right)100$$

In Equation 1, i indicates the percentage of occurrence of an event q in a universe of t events.

## Results

**Figure 1:**
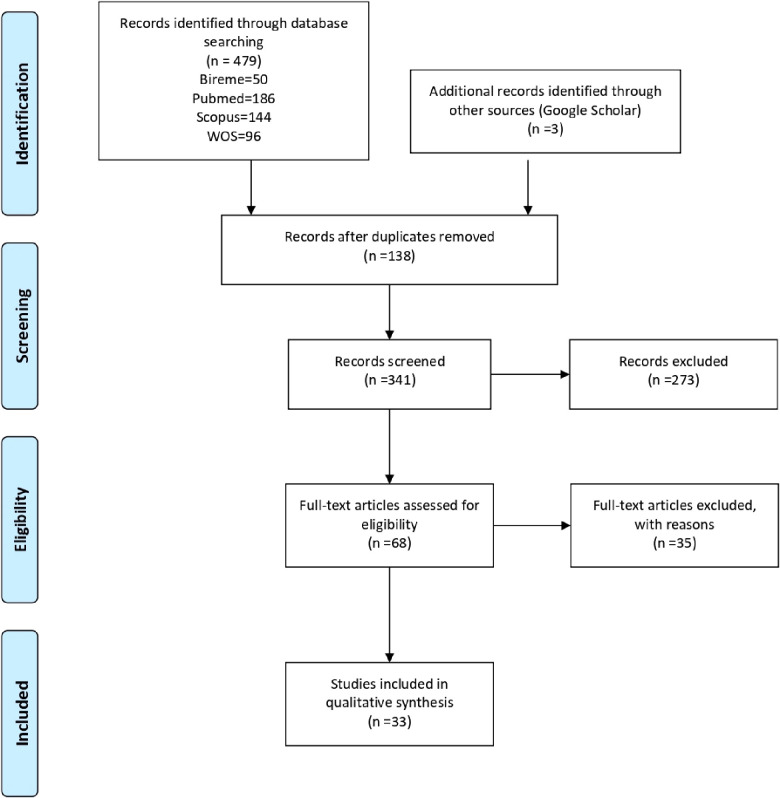
PRISMA-Scr flowchart.

A total of 479 studies were identified, of which 131 were duplicates. Based on the title and abstract, 341 studies were evaluated, 273 of which were excluded and 68 studies were evaluated at the full-text stage. For this scoping review, 33 studies were included, as detailed in **Supplementary Material 2**. Studies that did not meet the eligibility criteria were excluded in this last step. The flowchart according to PRISMA-Scr of the studies can be seen in Figure 1.

The included studies were published between the years 2010 and 2022. The countries of performance were: United States of America (n=18), England (n=6), France (n=4), Canada (n=1), Ireland (n=1), Belgium (n=1), India (n=1) and Japan (n=1).

### Characteristics of the included studies

Of the 33 articles included in this survey, twelve studies analyzed relevant aspects and good practices in translational research, such as solutions that integrate the technical and scientific perspectives and include aspects of commercial translation and regulatory requirements (36–47). Of these eleven, six studies involved in their research strategic actors of the local innovation system or of a specific segment. Numata et al. (46) studied the development of innovation in medical devices in Japan. Horgan and Lal (41) analyzed the basis for formulating personalized medicine strategy for the European Union - EU. Bayon et al. (37) formulated strategies focusing on the biomaterials segment. Bano et al. (36) addressed the relevant points and bottlenecks of translational research in India. Letourneur et al. (43) analyzed the ability of European universities to attract investments to meet the regulatory demands of translational research. Tromberg et al. (39) presented strategies for the area of biophotonics and biomedical optics. Another six articles addressed case studies and literature review that identified good practices and management tools that can contribute to the development of translational research (40,42,44,47–49). Fernandez-Moure (40) addressed the need to break the traditional concepts of disciplinary models, highlighting the importance of significant changes in academic training in this context. Marcus et al. (48), pointed out the importance of clinical collaboration between different specialists and other healthcare professionals in the success rate of innovative medical devices. Lacey and Sutherland (42) warned about the observance of the specificity of some technologies that do not fit the translational model pattern, due to their importance to the local and global community, for example, the technological ecosystem for tackling Covid-19 used in the Brazilian Unified Health System (26). Linehan and Chaney (44) developed their studies in offering mentorship as an effective strategy to meet defined milestones throughout the development process of a medical device. Vecht et al. (38) addressed the need for market potential analysis, prototyping, and testing, and support to researchers on technical, regulatory, and intellectual property issues. Lottes et al. (47) advocated prior submission of development projects to regulators and funding agencies as a useful strategy to anticipate requirements and thus better manage research risks.

New approaches or conceptual models applied to the development of translational research in medical devices were explored in nine articles (50–58).Borton et al. (50) highlights collaborative and coordinated models involving the community, industry, and academia to accelerate the process of translational research in neurology. Schwartz and Macomber (52) provided a roadmap, with an overview and structure, from three pillars: concise and validated problem definition, conceptual health or innovation model, and risk management. Of these nine surveys, two focused on collaborative, multidisciplinary, or innovative consortia approaches that considered the expertise of industry, academia, and clinical practitioners as a determining factor in advancing translational research (51,53). Sanami et al. (51) used the stages of the Gartner Hype Cycle, which provides a graphical representation of product and technology maturity and adoption, and Bayon et al. (53) the conception of value generation in healthcare. Two other studies made explicit in their models the focus on the user’s needs of the service or technology, from the early stages of the translation process (53,56). One study incorporated the instance of government in the structure of the model, intending to extend the scope and level of social impact, however, it considered a limited set of public resources for this purpose (55). Marcus et al. (57) presented a translational model driven by the risk management of the device under investigation. The model proposed by Lane (55) starts from the Knowledge to action (KTA) approach, which addresses the transformation of knowledge into behavioral change, and progresses to the Need to Knowledge (NTK) model (55), based on the finding that the necessary knowledge is not fully available. The model proposed by Kleinbeck et al. (56) drew attention to the need to incorporate critical competencies into undergraduate and graduate education, in synergy with Fernandez-Moure (40), who addresses the need for change in academic thinking that is occult to the disciplinary model.

Six studies analyzed institutional programs structured to overcome barriers to the translation of medical devices research, from the technical, scientific, and regulatory support (59–63). Pitzen et al. (59) evaluated the results of the Transform the Practice - Mayo Clinic Program after five years, when 24 teams of the program received resources and the involvement of senior management in removing barriers. In his study, the author highlights that, about 75% of the teams achieved at least one favorable outcome, such as advancing through the phases of a clinical trial (29%), obtaining a patent (25%), implementing a new line of care or service (25%), and developing a new diagnostic test (33%). Carleton et al. (64) analyzed the results of the multi-center Point-of-Care Technology Research Network (POCTRN) scientific network from 2012 to 2017, which supported more than 90 projects and provided financial resources, consulting, and engagement to nearly 2,500 researchers. Greenhalgh et al. (63) conducted an organizational case study at the United Kingdom Biomedical Research Centre. The latter research considered the principles of action research to support research themes and generate cross-disciplinary learning in a cross-cutting way. This study also addressed several subthemes: drug development, device development, business support and commercialization, research methodology and statistics, health economics, bioethics, patient and public engagement and involvement, knowledge translation, and education and training. Berro et al. (61) looked at how 24 academic health centers (AHCs) assist researchers with regulations and responsibilities. Their study identified that only half of the centers reported having a support office to conduct advanced training for researchers. Fischer et al. (60) outlined guidelines for a program with a mission to develop medical technologies for pediatric patients and contribute to the national innovation effort for this segment. Leuthardt (62) evaluated the Neurotechnologies Centre - NIH results, which, in three years, hired 32 clinical and non-clinical researchers and obtained 47 innovative ideas and 12 patents, of which 7 have already been licensed to the industry. Pienta (65) analyzed the partnership program between the University of Michigan and the Coulter Foundation that provides a model of institutional support that benefits researchers through structured support.

Six articles implemented education and training programs, starting with integrating technical, clinical and regulatory knowledge in the development process of new medical devices (66–71). Miller et al. (71) developed a multimedia-based online course focusing on medical device design and regulation fundamentals. Ribeiro et al. (66) adopted a pedagogical model based on experiential learning, design thinking, and competency-based learning in a hybrid format, with courses offered on an e-learning platform on topics related to technological innovation in healthcare. Their research was followed by an immersive internship with an experience in a health care facility to promote observation and identification of problems and unmet health needs. A survey of solutions and requirements was contemplated, culminating with ideation sessions in multidisciplinary groups, with support and mentoring for the development of the research. Domschke and Blaho (67) described a proposal for a new master’s curriculum focused on product development in translational medicine, based on the Stage-Gate industrial process, which is characterized as a tool used by industries to manage complex development processes. Yazdi and Acharya (68) analyzed a master’s program focused on innovation and design in bioengineering whose goal is to train engineers in creating health technologies, both advanced technologies, and technologies in global markets with scarce resources. Thompson et al. (70) evaluated the Case Western Reserve University (CWRU) Training Program that funded researchers to translate fundamental research discoveries to impact patients from an experiential learning model.

**Figure 2:**
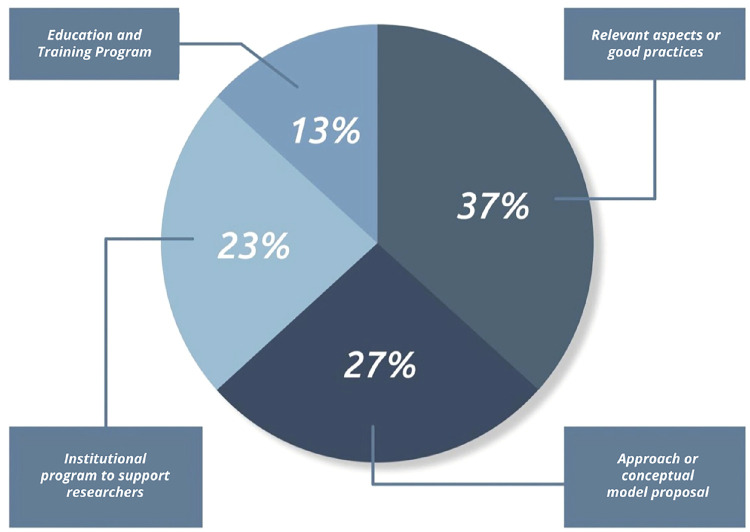
Classification of the included articles, by object of study.

The authors included in this review were dedicated to analyze translational research in medical devices from different perspectives and their distribution by object of study can be visualized in the chart in Figure 2.

### Knowledge, capacities and institutional structure for the development of TR

As already described in the topic of extraction and synthesis of data, for the systematization of the results extracted from the 33 articles included, three categories were adopted: characteristics, barriers faced and factors that favor translational research. The information was analyzed according to three dimensions, namely: knowledge, capabilities and institutional or inter-institutional structure.

From this approach, the characteristics of translational research in medical devices are described in Tables 1–3.

**Table 1:** Incidence of themes related to the attributes of translational research grouped by the Institutional dimension.

Dimension: Knowledge	Incidence N (%)
Regulatory technical (technical standards)	28 (85%)
Health regulatory (safety and efficacy)	27 (82%)
Knowledge about designing and conducting in vitro and in vivo assays	25 (75%)
Health Economics	22 (66%)
Regulatory Ethics	20 (60%)
Intellectual protection and asset management	21 (63%)
Knowledge of the conceptual model of the disease, problem area, workflow or process	18 (54%)
Product Life Cycle	16 (48%)
Product design	12 (36%)

Note: Articles grouped by barrier and dimensions according to incidence are available in **Supplementary Material 3**.

**Table 2:** Incidence of themes related to the attributes of translational research grouped by the capacity dimension.

Dimension: Capacity	Incidence N (%)
Collaborative Efforts	31 (94%)
Attention to unmet clinical needs	23 (70%)
Assessment of the technical, clinical and economic feasibility of the innovation	22 (69%)
Identification of promising technologies, at the frontier of knowledge and/or more cost effective	19 (58%)
Generation of value in healthcare	19 (58%)
Assessment of the technology’s market potential and business plan	21 (64%)
Effective communication with different stakeholders	19 (58%)
Tolerance to error and risk	15 (45%)
Use of project management tools and methodologies and management support	14 (42%)
Analysis of the technology’s framework in the risk class to identify technical and sanitary regulations and requirements	14 (42%)
Innovation project documentation containing milestones, timeline, requirements, and available resources	10 (30%)
Attention to product usability in the early stages, in order to increase the chances of success and allow for timely reorientation in development	9 (27%)
Entrepreneurship	4 (12%)
Creativity	4 (12%)

Note: Articles grouped by barrier and dimensions according to incidence are available in **Supplementary Material 3**.

**Table 3:** Incidence of themes related to the attributes of translational research grouped by the Institutional dimension.

Dimension: Institutional or inter-institutional structure	Incidence N (%)
Interdisciplinary and Transdisciplinary Teams	31 (94%)
Collaborative or networked action linking multiple researchers and research centers and the government	28 (85%)
Development of partnerships with industry	21 (64%)
Education and Training on pertinent topics and with appropriate educational resources	22 (66%)
Support to researchers in project management, monitoring and evaluation	20 (60%)
Support about available funding (Governmental agencies and non-governmental entities)	18 (54%)
Strategic and risk management support	15 (45%)
Administrative, legal and/or regulatory support	14 (42%)
Prototyping in industrial design based on clinical and user needs assessment	14 (42%)
Proof of concept, prototype and in vitro testing capabilities	13 (39%)
Mentoring and supervision	11 (33%)
Adoption of business and marketing strategies	11 (33%)
Opportunity for start-up creation, technology transfer or licensing	10 (30%)
Adoption of good laboratory practices (GLP) and quality management	7 (21%)
In-house or collaborative clinical research infrastructure	6 (18%)
Engaging institutional leaders to remove technical and administrative barriers	6 (18%)

Note: Articles grouped by barrier and dimensions according to incidence are available in **Supplementary Material 3**.

As evidenced in Tables 1–3, the contents pointed out as fundamental for the development of translational teams include technical (85%), sanitary (82%), ethical (60%) and patent (63%) regulations. Knowledge of techniques for designing and conducting in vitro and in vivo trials (76%) and understanding the conceptual model of the disease (54%), such as lines of care, protocols, and therapeutic guidelines, are also pointed out as well as fundamental. Knowledge of Health Economics, such as the cost-effectiveness of technologies, budgetary impact, financing and payment issues, organization, and parameters adopted for incorporating technologies in the health system, are pointed out in 66% of the articles.

The studies indicate essential capabilities for developing translational research, such as the need for collaborative efforts (94%) and merging scientific and technological responses with unmet clinical conditions (70%). The ability to assess the technical, clinical and economic feasibility of the innovation (69%) and management support tools (42%), capable of anticipating obstacles are considered essential skills for the translational research process. Also mentioned is the development of environments that are more tolerant to error and risk (45%) and promote more effective communication (58%) between the actors involved.

Most studies also point to the need for attention to models that enable the generation or co-creation of value in health (58%), from the transformation of discoveries into innovations with social impact. In contrast, only 12% of the studies mention the need to develop creative and entrepreneurial skills.

It was also clear that the challenge for the conformation of institutional and/or inter-institutional structures that promote fertile environments for translational research comprises the structuring of interdisciplinary and transdisciplinary teams (94%) that act in a collaborative and networked way (85%) and the development of partnership with industry (64%). Another aspect evidenced is the need to offer training (66%) and institutional support in technical and regulatory issues (60%).

The barriers faced by academic laboratories in the process of translating medical device research are highlighted in Table 4.

**Table 4:** Incidence of the barriers pointed out as determinants to be faced by academic laboratories in the implementation of translational research, from the analysis dimension.

Barriers	Dimension			Incidence N (%)
	Kno	Cap	IS	
Untrained staff	∙	∙		24 (73%)
Lack of institutional support for the teams			∙	21 (64%)
Inadequate financing			∙	20 (60%)
Institutional model and public policies based on disciplines and linear innovation process			∙	19 (57%)
Difficulty in interacting with industry and society		∙	∙	18 (54%)
Difficulty in communication		∙	∙	16 (48%)
Researcher Training Programs	∙	∙		15 (45%)
High cost of the research translation process and patent maintenance			∙	11 (33%)
Long time to complete the entire translation process	∙	∙	∙	12 (36%)
Industry disinterest			∙	5 (15%)
Lack of agility in the university’s administrative processes			∙	3 (9%)

Abbreviations: Kno, knowledge; Cap, capacity; IS, institutional structure.

Note: Articles grouped by barrier and dimensions according to incidence are available in **Supplementary Material 3**.

As shown in Table 2, the obstacles faced by academic laboratories in the research translation process include inadequate or deficient training of researchers (73%) and, to a large extent, institutional and governmental difficulties in conducting innovation in health, such as lack of funding (68%) and support for teams (64%). Outdated funding and action models (63%) and difficulties in interacting with industry and society (58%) are referred to as relevant barriers, with communication being the main factor for this lack of integration by about 48% of the studies.

In addition to the aspects mentioned, the determining factors for accelerating the medical device development process are shown in Table 5.

**Table 5:** Incidence of factors indicated as determinants for favoring the implementation of translational research, from the dimension of analysis.

Factors	Dimension			Incidence N (%)
	Kno	Cap	IS	
Multidisciplinary, collaborative, and networked teamwork		∙	∙	31 (94%)
Team skilled in technical and regulatory requirements	∙			28 (85%)
Cooperation with the industry		∙	∙	24 (73%)
Qualified and effective communication between the actors involved		∙	∙	21 (64%)
Valuing the patient’s and technology user’s perspective		∙	∙	19 (57%)
Clinical immersion to identify and assess unmet clinical problems and needs		∙	∙	14 (46%)
Offering regular funding to maintain the team			∙	14 (46%)
Changing the paradigm of researcher training			∙	14 (46%)
Selection of Projects with high translation potential		∙	∙	14 (46%)
Project Management		∙	∙	14 (46%)
Alignment of innovation with institutional and local priorities			∙	10 (30%)
Transparency with the aim of improving the quality of research and avoiding duplication of effort			∙	5 (15%)
Sustainability (effective technologies developed for low-resource environments)	∙	∙	∙	4 (12%)

Abbreviations: Kno, knowledge; Cap, capacity; IS, institutional structure.

Note: Articles grouped by barrier and dimensions according to incidence are available in **Supplementary Material 3**.

To accelerate the development of technologies and reduce the risks and uncertainties of the innovative process in health, interdisciplinary, transdisciplinary, collaborative and networked work (94%), the qualification of communication (64%), clinical immersion (46%), as well as valuing the perspective of the patient and the user of the technology (57%) and the regularity of financial resources contribution (46%) are pointed out as determining factors. Most articles also mention the need for change in the researchers training (85%), so that it reflects the future needs of a rapidly evolving health care system. Some studies also address the alignment of innovation with institutional and local priorities (30%) as a determining factor for the success of translational research. Transparency, aiming to improve the quality of research and avoid duplication of effort (13%), and sustainability (10%). However, we understand that these last three factors are essential for universal health systems, as in the Brazilian case, since the technologies developed will have to be effective, considering the population’s needs and environments with great demands and few financial resources.

## Discussion

Development in translational health research can contribute to facing today’s social and economic challenges, particularly in countries like Brazil, which has a Unified Health System (called SUS) and imports most of the technologies used. The balance of trade in the health sector, which was already in deficit, was strongly impacted by the Covid-19 pandemic, and presents a deficit of US$ 15 billion (18). It reveals a worrisome dependence on the foreign market for strategic technologies for the Brazilian health system, a critical scenario that has already been reported in recent years. Thus, it faces the need to adopt structural measures that raise the level of development of technologies conceived, arising from “know-how,” and that strengthen the productive base of the country.

The analysis of the articles included in this review points out key elements for developing “know-how,” from the translational research approach. Among them, we highlight the need for expansion and consolidation of knowledge from various areas, the development of capabilities, greater use of tools, and relevant partnerships to design and develop technologies that can be effectively incorporated and applied in the Brazilian health system. The effective use of technologies, that is, the translation of research, implies the capacity to meet clinical and management needs that strain the health system, that is, mission-oriented innovation, local or national problems (72). Therefore, in addition to the essential knowledge for a translational research team (regulatory, technical, ethical, patent, and sanitary), the understanding of local problems and needs, of the disease model or the process in which the technology will be inserted in the health system stands out as a differential. This implies knowledge about lines of care, protocols, and therapeutic guidelines, as well as the parameters adopted for the incorporation of technologies into the health system and issues related to the financing and payment model. As for the skills required of the translational researcher, they focus on the development of communication, in a way that favors the effectiveness of scientific collaboration with other researchers and with other institutions. As institutional skills, we highlight the need to offer support to the researcher and training in management tools, capable of monitoring stages and anticipating obstacles, as well as the development of environments that are more tolerant to error and risk and that promote more effective communication between the actors involved.

The development of health technologies is strongly regulated in Brazil. This characteristic, also evidenced in other countries analyzed in the included articles, gives translational researchers a kind of route that, if followed during the development process of a new device, minimizes relevant risks. It is critical that an understanding of this critical path of translational research be incorporated into the culture of universities and research centers in order to facilitate meeting a set of requirements, accelerate the process, and minimize the risks inherent in the development of innovative technologies. Thus, we understand that it is essential that the technical and regulatory route domains a requirement for funding agencies to fund translational research. This strategy can induce qualification in the area and increase the success rate of funded research.

The development of these critical competencies throughout the research translation process is essential for the conception of health technologies. Especially, because there is an accelerated process of digital transformation resulting from the 4th industrial revolution that is already ongoing and was promoted as a strategy to face the Covid-19 pandemic. The adoption of these disruptive innovation technologies challenged regulatory and management healthcare structures and will require the development of new competences. In Brazil, as a response to these challenges, normative efforts have been recently undertaken, in order to provide legal certainty and a favorable environment for the technological route development of the digital revolution, such as the publication of Anvisa Resolution (RDC 657/2022) (73), which regulates software as medical devices, and the regulation of Telemedicine (Resolution CFM 2,314/2022) (74).

However, a favorable regulatory environment and a solid knowledge base are not enough to promote changing behavior and the adoption of best practices in the development of innovative health technologies. Improvement of practice occurs through systematic reflection on action, planning, implementation, description, and evaluation to expand knowledge throughout the process. The realization that in the development of innovative technology, the necessary knowledge is not fully available makes us reflect on the need to implement institutional models that are more efficient in promoting multidisciplinary collaboration and institutional and inter-institutional cooperation. This need is especially critical in healthcare, which comprises a broad set of products and processes associated with clinical practices, institutional arrangements, management support solutions, treatment protocols, drugs, vaccines, and medical devices. The institutional model cannot be restricted to the development of tangible technologies and must consider the specificities of non-tangible technologies, which may not fit into the standard translational model due to their characteristics, and require different strategies to be incorporated and disseminated.

Translational research can be better understood in the Brazilian innovation system and the CEIS, since it is favorably impacted by improved interaction between local actors (university, health services, government, and industry). In this approach, the formulation of public policies for promotion and regulation and the development and continuity of instances of articulation are strategic for the development of translational research. These instances, if well-coordinated, can contribute to the convergence between sectoral public policies of health, science, technology, and innovation and economic and industrial development, with local and national needs, academic competencies, and the national productive capacity. This coordinated effort may be the path to developing translational research and the local production of technologies strategic to the country.

## Conclusion

It was observed the adoption of a common assumption in all the studies analyzed: the need for an interdisciplinary, transdisciplinary and collaborative approach for the development of translational health research. This approach aims to solve real problems, socially relevant and whose solutions are beyond the scope of a single discipline or research area.

Furthermore, the need for integration between various actors (researchers and institutions) was identified as an indispensable condition for translational research to be understood in the framework of the Brazilian innovation system and the CEIS. This is because the involvement of these actors is a fundamental contribution of translational research for the implementation of public policies and institutional models that produce fertile conditions for the generation and diffusion of innovations in health.

The dimensions adopted in this scoping review provided the identification and systematization of the characteristics and factors that integrate translational research in medical devices, consequently helping researchers and managers to clarify the theme and its application in health. The reflection on the challenges and barriers to the implementation of translational research serves as a basis for adopting institutional strategies and the formulation of public policies aimed at developing integrated solutions that the health system can incorporate.

As limitation of this review, it is highlighted that the non-inclusion of Brazilian studies and the greater concentration of studies in developed countries, which do not have a universal health system. This reveals the need for future primary studies and reviews to ensure that other important results are evaluated and contribute to the understanding of the Brazilian context. This review also challenges the development of future research dedicated to the analysis of aspects of digital transformation, advances in the regulatory framework and the impact of this new context on the development of competencies for the translation of health research.

## Data Availability

The original contributions presented in the study are included in the article/**Supplementary Material**, further inquiries can be directed to the corresponding authors.
