# Supplementary material for: Translational research in health technologies: A scoping review
Source: Front Digit Health. 2022 Aug 3;4:957367. doi: 10.3389/fdgth.2022.957367 (PMC9385029; doi:10.3389/fdgth.2022.957367)
Supplement: Supplementary file 1 [file Data_Sheet_1_v1.pdf]

Supplementary Material 1

1 Search Strategy

Initial search date: 28/11/2021

Search update: 29/04/2022

BIREME:

mh:"Pesquisa Médica Translacional" OR (Investigaciónen Medicina Traslacional) OR (Translational Medical Research) OR (Pesquisa Translacional) OR mh:H01.770.644.145.675$ AND mh:"Equipamentos e Provisões" OR (Equipos y Suministros) OR (EquipmentandSupplies) OR(Dispositivo) OR (Dispositivo Médico) OR (Dispositivos) OR(Dispositivos Médicos) OR (Equipamento) OR(Equipamentos)OR mh:E07$ OR SP4.006.062$ OR VS2.006.001$ AND mh:"Projetos de Desenvolvimento Tecnológico e Inovação" OR (Proyectos de Desarrollo Tecnológico e Innovación) OR (TechnologicalDevelopmentandInnovationProjects) OR mh:SH1.020.030.020$ OR mh:"Inovação" OR (Innovación) OR (Innovation) OR mh:SH1.020.010.090$

PUBMED:

(("Translational Medical Research"[Mesh] OR (Medical Research, Translational) OR (Research, Translational Medical) OR (Translational Medical Science) OR (Medical Science, Translational) OR (Medical Sciences, Translational) OR (Science, Translational Medical) OR (Sciences, Translational Medical) OR (Translational Medical Sciences) OR (Translational Research, Medical) OR (Medical Translational Research) OR (Research, Medical Translational) OR (Translational Medicine) OR (Medicine, Translational) OR (Knowledge Translation) OR (Knowledge Translations) OR (Translation, Knowledge) OR (Translations, Knowledge) OR (Translational Research) OR (Research, Translational) OR (Translational Researches)) AND ("Equipment and Supplies"[Mesh]OR (Supplies and Equipment) OR (Apparatus and Instruments) OR (Instruments and Apparatus) OR (Medical Devices) OR (Medical Device) OR (Device, Medical) OR (Devices, Medical) OR (Devices) OR (Device) OR (Equipment))) AND ("Inventions"[Mesh] OR (Invention) OR (Technological Innovations) OR (Innovation, Technological) OR (Innovations, Technological) OR (Technological Innovation))

SCOPUS:

(TITLE-ABS-KEY ("Translational Medical Research" OR "Medical Research, Translational" OR "Translational Research" OR "Translational Medical Science" OR "Research, Translational Medical" OR "Medical Sciences, Translational" OR "Science, Translational Medical" OR "Translational Medical Sciences" OR "Medical Translational Research" OR "Translational Medicine" OR "Knowledge Translation") AND TITLE-ABS-KEY ("Medical Devices" OR "Medical Device" OR equipment OR "Supplies and Equipment" OR "Apparatus and Instruments" OR devices OR device) AND TITLE-ABS-KEY (invention OR innovation OR "Technological Innovation" OR "Technological Innovations" OR "Innovation, Technological" OR "Innovations, Technological"))

Web Of Science:

(Translational Medical Research* OR Translational Research* OR Translational Medical Science* OR Medical Translational Research* OR Translational Medicine* OR Knowledge Translation*) AND TÓPICO: (Medical Devices* OR Medical Device* OR Equipment* OR Supplies and Equipment* OR Apparatus and Instruments* OR Devices* OR Device*) AND TÓPICO: (Invention* OR Innovation* OR Technological Innovation* OR Technological Innovations*)

Indexes: SCI-EXPANDED, SSCI, A&HCI, CPCI-S, CPCI-SSH, BKCI-S, BKCI-SSH, ESCI, CCR-EXPANDED, IC.
